# Megacystis microcolon intestinal hypoperistalsis syndrome

**DOI:** 10.11604/pamj.2018.31.109.16702

**Published:** 2018-10-12

**Authors:** Nishat Fatema1, Houda Nasser Al Yaqoubi

**Affiliations:** 1 Department of Gynaecology & Obstetrics, Ibri Regional Hospital, Ministry of Health, Sultanate of Oman

**Keywords:** Distended bladder, mild polyhydramnios, fetal bilateral hydronephrosis

## Image in medicine

A 34-year old gravida03 para02 woman with fetal bilateral hydronephrosis (A), greatly distended bladder and mild polyhydramnios, detected during a prenatal ultrasound. Based on the ultrasonography findings, megacystis microcolon intestinal hypoperistalsis syndrome (MMIHS) was suspected. MMIHS is an unusual disorder with the incidence of 1:1500, characterized by a massively enlarged fetal urinary bladder, microcolon, hypoperistalsis throughout the intestinal tract and incomplete intestinal rotation. A female infant was born at 34 weeks of gestation by an emergency caesarean section because of breech presentation during labor. The neonate weighed 4.2 kg with an Apgar score of 4 in 1 min and 6 in 5 min. Physical examination showed massive distended abdomen (B) and bladder, as well as bilateral palpated enlarged kidneys. Abdominal plain x-ray showed a dilated stomach and minimal gas in the distal bowel segments (C). An abdominal ultrasound imaging showed dilated stomach with distal duodenal atresia. Fluoroscopy gastrografin revealed narrow bowel loops and jejunal malrotation at the right side. Kidney-urinary-bladder ultrasonography showed distended urinary bladder, bilaterally enlarged kidneys, with tortuous and dilated ureter. A vesicostomy was performed to facilitate the drainage of urine (D). Presence of ganglion cells on rectal biopsy excludes Hirschsprung disease. The condition is fatal and surgical correction often remained unsuccessful. MMIH is an autosomal recessive disorder so genetic counseling is suggested for future pregnancies.

**Figure 1:**
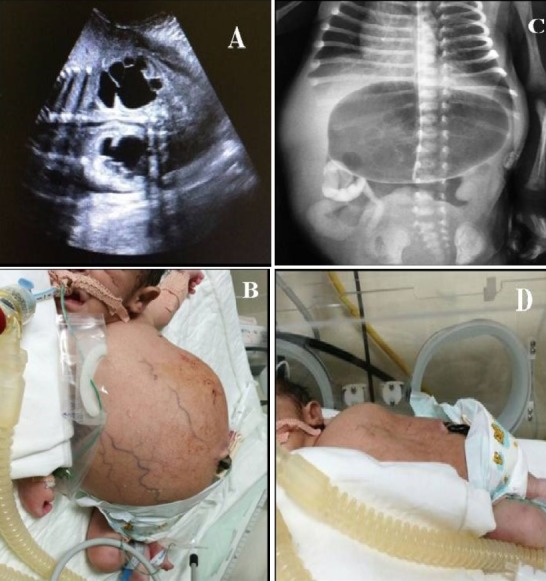
A) antenatal ultrasonography shows fetal bilateral hydronephrosis; B) massive distended abdomen secondary to distended urinary bladder; C) abdominal X ray shows dilated stomach; D) after vesicostomy, reduced abdominal distension

